# Peripheral retinal neovascularization secondary to highly myopic superficial Retinoschisis: a case report

**DOI:** 10.1186/s12886-020-1308-6

**Published:** 2020-01-13

**Authors:** Mingyue Luo, Hong Du, Hua Ding, Rongping Dai

**Affiliations:** 1 Department of Ophthalmology, Peking Union Medical College Hospital, Chinese Academy of Medical Sciences, Beijing, 100730 China; 2 Key Laboratory of Ocular Fundus Diseases, Peking Union Medical College, Chinese Academy of Medical Sciences, Beijing, China; 3 Department of Ophthalmology, Datong Coal Mine Group Hospital, Datong, 037003 Shanxi China

**Keywords:** Pathological myopia, Retinoschisis, Retinal microvascular abnormalities, Retinal neovascularization

## Abstract

**Background:**

Peripheral Retinal neovascularization is well-described as a complication of X-linked retinoschisis, but less often observed in myopic and primary retinoschisis. We present a case of a myopic female who developed retinal microvascular abnormalities due to retinoschisis and subsequent vitreous hemorrhage which would cause severe visual damage without timely and proper treatment.

**Case presentation:**

A 38-year-old highly myopic Chinese female complained of blurred vision in her right eye. Her best corrected visual acuitiy was 20/20 OU, and her refraction was − 9.00S OU. Dilated fundus examination revealed mild vitreous hemorrhage and abnormal vascular network nasal to the optic disc in her right eye. Optical Coherence Tomography (OCT)- angiography (OCTA) B-Scan showed superficial retinoschisis and well-depicted abnormal retinal microvascular network in inner retinal layer. Sectoral scatter laser photocoagulation was administered. Regression of most abnormal vessels was achieved in 1 month, but the patient experienced an unexpected episode of vitreous hemorrhage 3 months after the initial treatment, which was absorbed spontaneously in 2 weeks. Supplemental laser photocoagulation was applied and regular follow-up visit was suggested.

**Conclusion:**

Superficial retinoschisis in pathological myopia can be a driver of retinal microvascular abnormalities, possibly neovascularization, an extremely rare but severe complication which can be vision-threatening without timely and proper intervention.

Peripheral Retinal neovascularization is well-described as a complication of X-linked retinoschisis [1], but less often observed in myopic and primary retinoschisis. There are some cases reported as retinal neovascularization [2] or microvascular abnormalities [3], yet their nature remains debatable. In this report, we present a case of retinal microvascular abnormalities, possibly neovascularization and subsequent vitreous hemorrhage secondary to highly myopic superficial retinoschisis. This report was organized in adherence to CARE guidelines.

## Case presentation

A 38-year-old highly myopic female presented with blurred vision and floaters in her right eye to the ophthalmology department in December, 2018. No ocular or systemic medication was reported. Her corrected visual acuity was 20/20 OU, and her refraction was − 9.00S OU. The anterior segments were normal in both eyes.

**Fig. 1:**
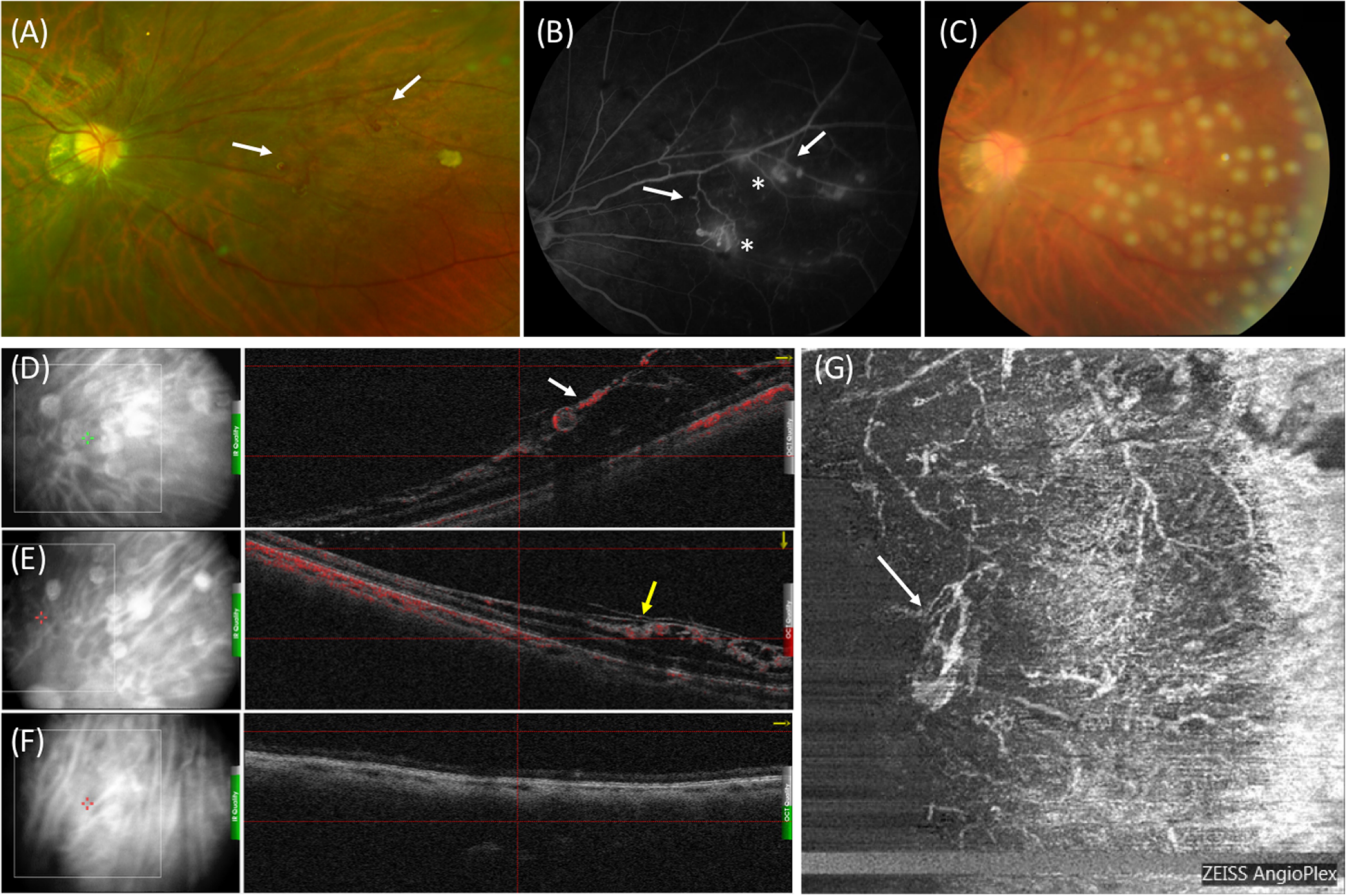
Multi-model imaging applied to the patient in her first visit. **a** Scanning laser ophthalmoscopy (SLO) showing fine, tortuous inter-connecting vascular network (white arrows) in the superficial layer of the retina nasal to the optic disc in her right eye. **b** Fluorescein fundus angiography (FFA) showing abnormal aneurysmal vessel network (white arrows) with obvious fluorescein leakage (asterisk marks). **c** Color fundus photography taken immediately after sectoral scatter laser photocoagulation. **d** and **e** Well-depicted retinal neovascularization at the level of retinal nerve fiber layer (NFL, white arrow) and ganglion cell layer (GCL, yellow arrow) overlying retinoschisis in optical coherence tomography angiography (OCTA) B-Scan. **f** Normal OCT B-Scan in other quatrants. **g** En-face OCTA showing fine inter-connecting vascular network (white arrow)

Dilated ophthalmoscopic examination revealed mild vitreous hemorrhage and abnormal vascular network nasal to the optic disc in her right eye. There were no pars plana exudates with snowbanking or inferior snowballs. Scanning laser ophthalmoscopy (SLO) showed fine, tortuous inter-connecting vascular network in the superficial layer of the retina (Fig. 1a). Fluorescein fundus angiography (FFA) in her local hospital showed abnormal aneurysmal vessel network with obvious fluorescein leakage (Fig. 1b). Optical Coherence Tomography (OCT)- angiography (OCTA) B-Scan showed superficial retinoschisis and well-depicted abnormal vessels in the inner retinal layer (Fig. 1d-g). Neither retinoschisis nor vascular abnormalities were detected in other quadrants. Her left eye was relatively normal in all the aforementioned examinations.

**Fig. 2:**
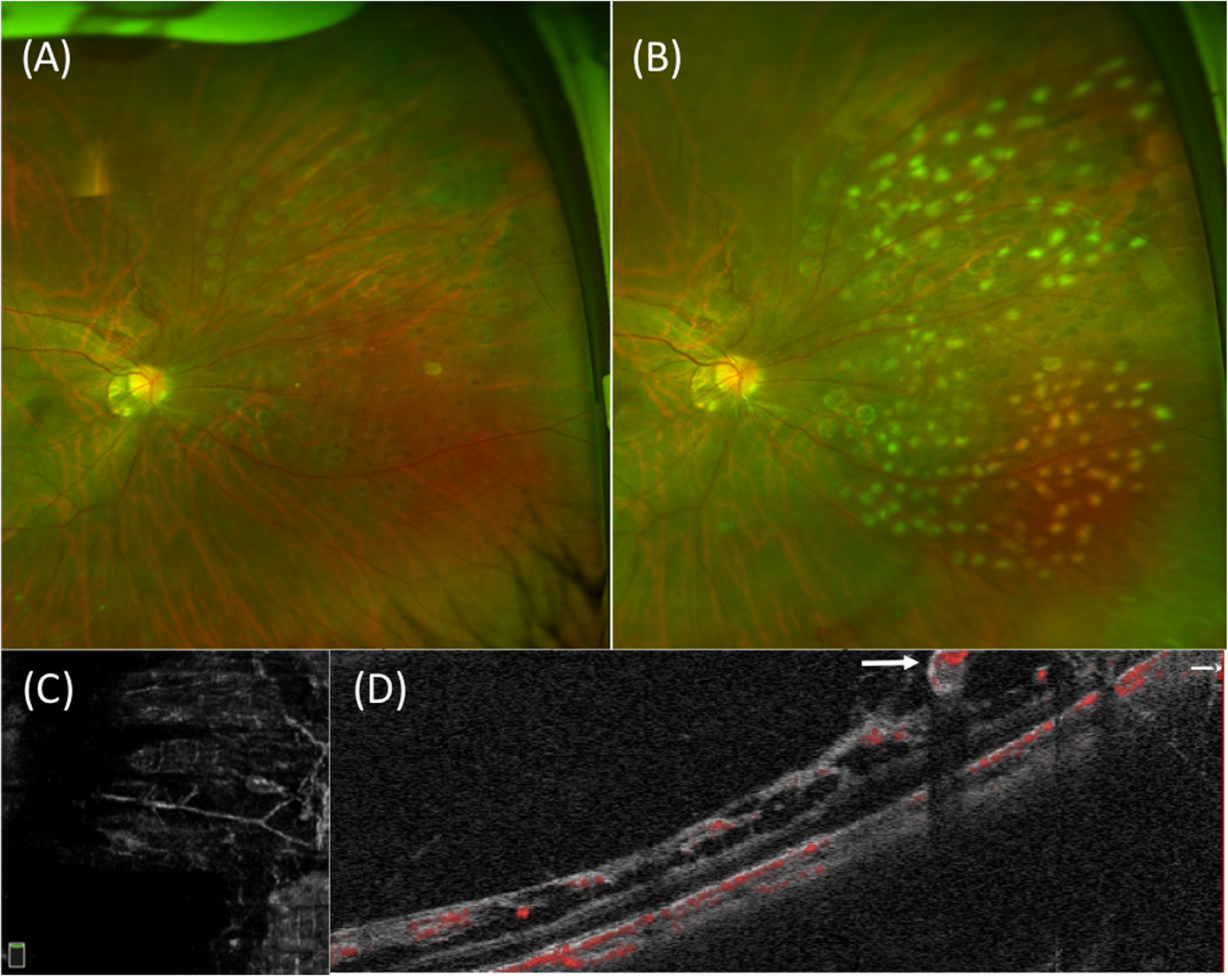
Ophthalmic imaging of follow-up visit. **a** Regression of most retinal neovascularization confirmed by scanning laser ophthalmoscopy (SLO) during the 2nd visit. **b** SLO image after supplemental laser coagulation in the 3rd visit. **c** and **d** Remaining retinoschisis and aneurysmal structure (white arrow) showed by en-face optical coherence tomography angiography (OCTA) and OCTA B-Scan in the 3rd visit

Sectoral scatter laser photocoagulation was administered since the retina was clearly visible and out of economic reasons. Color fundus photography was performed immediately (Fig. 1c). Regression of most abnormal vessels was confirmed by SLO in her second visit 1 month later (Fig. 2a), but an unexpected episode of vitreous hemorrhage obscuring the posterior pole was found 3 months later with her BCVA decreased to 20/40, which was absorbed spontaneously in 2 weeks and BCVA back to 20/20. OCTA B-Scan showed retinoschisis and the aneurysmal structure remained relatively stable despite regression of retinal microvascular abnormalities (Fig. 2c-d). Supplemental laser photocoagulation was applied and regular follow-up visit was suggested (Fig. 2b).

## Discussion and conclusions

Peripheral vascular abnormalities often passed undiagnosed unless causing clinically significant symptoms, mostly related to vitreous hemorrhage due to rupture of retinal vessels [1]. In this case, the vitreous hemorrhage blurred the patient’s vision, and made it possible for a timely diagnosis.

Retinal microvascular abnormalities secondary to retinoschisis has been reported in some cases, especially in X-linked retinoschisis (XLRS), and often referred to as retinal neovascularization [4]. But these retinal changes are relatively less observed in myopic and primary retinoschisis [1]. The notion of ischemia-induced neovascularization is supported by cases of abnormal vessels distant from the lesions, such as iris [5] and optic disc [1]. Others reported cases of microvascular abnormalities with similar appearance to neovascularization, which were considered as compensation for the schitic process instead. Durkin et al. [3] reported a case of retinal microvascular abnormalities appearing to be “dilated capillary terminals” without fluorescein leakage secondary to myopic retinoschisis, and hypothesized that capillary remodeling occurred as the inner and outer retinal leafs separated, which may induce bleeding if the schitic cavity progressed more rapidly, mimicking leakage of neovascularization in FFA. Ong et al. [6] reported retinoschisis-related telangiectatic retinal vessels or retinal aneurysmal dilatations in the absence of capillary non-perfusion in OCTA, disputing ischemia-induced neovascularization. It remains debatable whether these abnormalities are vascular remodeling merely due to retinoschisis or related to ischemia-induced neovascularization, or somewhere in between.

In our case, we think ischemia plays a more significant role. Firstly, the abnormal vessels are very superficial depicted by OCTA, thus unlikely to be vessel remodeling bridging inner and outer schitic layers. Secondly, the hyperfluorescence in FFA is more likely to be leakage from vessel walls instead of discharging from ruptured vessel terminals. Lastly, a single sectoral laser wasn’t able to stabilize patient’s condition, resulting in recurrent vitreous hemorrhage. Specifically, we would like to stress the role of superficial retinoschisis in the initiation of neovascularization process. OCT images of similar cases were reviewed in myopic and primary retinoschisis in patients, and we found that the schisis mostly involved RNFL and/or GCL, with deeper layers involved in some cases [2, 3, 6] We hypothesized that ischemia is more prominent in superficial retinoschisis, as loss of blood supply due to tear of superficial capillary plexus (SCP) could render the inner retinal layer almost completely ischemic, which triggers a molecular cascade and subsequent retinal neovascularization elicited by vascular endothelium growth factor (VEGF). Unfortunately, since the lesion was not at a routine location for our OCTA scan and the quality of FFA from her local hospital was not high enough to visualize non-perfusion area, we couldn’t rule out either retinoschisis-related vascular remodeling or ischemia-induced neovascularization.

Limitations of this report include the retrospective nature and small sample. Yet our case reinforces the necessity of a thorough examination in myopic patients. We reported and summarized retinal microvascular abnormalities, possibly neovascularization as a severe complication of superficial retinoschisis, which can be vision-threatening without timely and proper intervention.

## Data Availability

Not applicable.

## Abbreviations

FFA: fluorescein fundus angiography
GCL: ganglion cell layer
OCT: optical coherence tomography
OCTA: optical coherence tomography angiography
PM: pathological myopia
RNFL: retinal nerve fiber layer
SCP: superficial capillary plexus
SLO: Scanning laser ophthalmoscopy
VEGF: vascular endothelium growth factor
XLRS: X-linked retinoschisis
